# Determining glioma cell invasion and proliferation in *ex vivo* organotypic mouse brain slices using whole-mount immunostaining and tissue clearing

**DOI:** 10.1016/j.xpro.2022.101703

**Published:** 2022-09-21

**Authors:** Jessy V. van Asperen, Emma J. van Bodegraven, Pierre A.J.T. Robe, Elly M. Hol

**Affiliations:** 1 Department of Translational Neuroscience, UMC Utrecht Brain Center, University Medical Center Utrecht, University Utrecht, Utrecht, The Netherlands; 2 Department of Neurology and Neurosurgery, UMC Utrecht Brain Center, University Medical Center Utrecht, University Utrecht, Utrecht, The Netherlands

**Keywords:** Cell biology, Cancer, Microscopy, Antibody, Neuroscience

## Abstract

The *ex vivo* organotypic brain slice invasion model is commonly used to study the growth dynamics of gliomas, primary brain tumors that are known for their invasive behavior. Here, we describe a protocol where the *ex vivo* organotypic mouse brain slice invasion model is combined with whole-mount immunostaining, tissue clearing, and 3D reconstruction, to visualize and quantify the invasion of glioma cells. In addition, we describe an approach to determine the proliferation rate of the cells within this model.

For complete details on the use and execution of this protocol, please refer to Uceda-Castro et al. (2022).

## Highlights

• Preparation of *ex vivo* organotypic brain slices and injection with glioma cell lines
• Whole-mount immunostaining protocol for the co-cultured brain slices
• Analysis workflow to quantify glioma cell invasion using ImageJ and Imaris
• Application and processing of a BrdU-assay of glioma cells in brain slice cultures

## Abstract (editor highlights)

Publisher’s note: Undertaking any experimental protocol requires adherence to local institutional guidelines for laboratory safety and ethics.

## Graphical abstract

**Figure:**
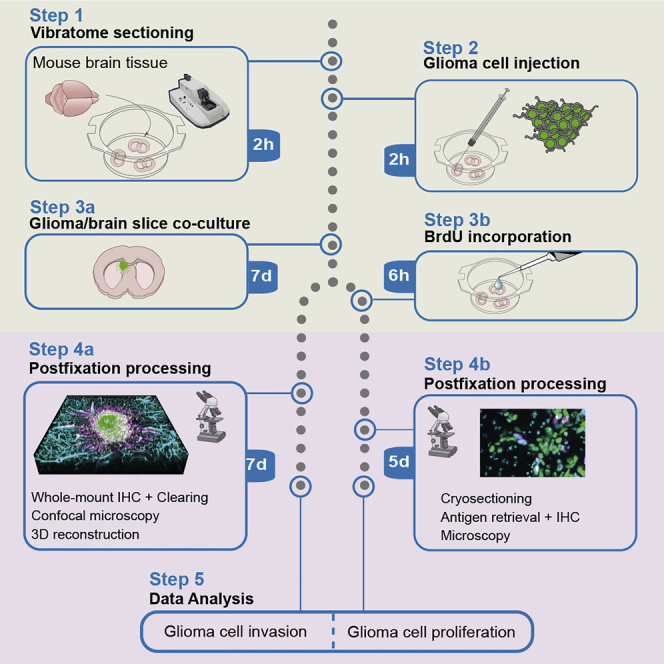

## Before you begin

### Institutional permissions

This protocol requires the use of mouse brain tissue. All animal experiments should be performed following relevant governmental and institutional guidelines. All experiments described here were approved by the Animal Welfare Body Utrecht and the Central Authority for Scientific Procedures on Animals of the Netherlands (CCD, license: AVD115002016532).

### Generation and expansion of fluorescently labeled glioma cell lines

**Timing: 10 days**

The protocol and analysis steps described in this paper are based on U251-MG cells that are stably expressing histone-2B (H2B)-mNeonGreen and/ or H2B-mCherry, such that the nuclei are fluorescently labeled. We have also used this protocol successfully with other glioma cell lines such as the human U87 glioblastoma line, the GL261 mouse glioblastoma line, and primary glioblastoma cell lines. Cytosolic- or membrane-bound fluorophores can also be used to visualize the glioma cells, however, the quantification steps described in this protocol are optimized for nuclear labeling. We do not recommend the use of fluorescent dyes for this protocol, as the fluorescent dye is lost during the clearing steps (steps 40 and 41).

**CRITICAL:** Production of lentiviruses and transduction of cells with lentivirus should be performed in a biosafety level 2 certified laboratory**.**

1. Production of lentivirus:
  a. On day 1, plate 2 × 10^7^ cells 293T/17 cells in 15 cm^2^ dishes, use two plates per lentiviral construct. Culture the 293T/17 in DMEM high glucose + 10% Fetal Bovine Serum (FBS) and 1% Penicillin-Streptomycin (P/S).
  b. On day 2, refresh the medium one hour before transfection.
  c. Prepare mix 1 (103.2 μg DNA in 1 000 μL DMEM high glucose medium, for two 15 cm^2^ plates):
    i. 45.0 μg 3^rd^ generation lentiviral vector.
    ii. 15.8 μg pMD2.G (VSV-G).
    iii. 31.2 μg pMDLg/PRRE.
    iv. 11.2 μg pRSV-Rev.
  d. Prepare mix 2 by adding 160 μL Polyethylenimine (PEI) to 1 000 μL DMEM high glucose medium, for two 15 cm^2^ plates.
  e. Mix transfection mixes 1 and 2 by vortexing and incubate at room temperature (19°C–21°C) for 15 min.
  f. Add the total transfection mix to the two plates with 297T/17 cells.
  g. On day 3, replace the medium with 17 mL per dish of DMEM high glucose with 2% FBS and 1% P/S.
  h. On day 4, collect the viral supernatant of the two dishes in a 50 mL tube. Spin the medium for 5 min at 300 × *g* and filter the supernatant through a 0.22 μm filter.
    ***Optional:*** The lentivirus can be concentrated using ultracentrifugation to increase the titer.
  i. Aliquot and freeze the virus suspension at – 80°C for long-term storage.
  j. Determine the virus titer using the Lenti-X GoStix Plus or by transduction of 293T/17 cells.
2. Transduction of U251-MG glioma cells.
  a. Plate 1 × 10^6^ cells per well in a 6-well plate (9.6 cm^2^). Culture the U251-MG cells in DMEM high glucose mixed 1 to 1 with F10 nutrient mix, with 10% FBS and 1% P/S.
  b. Twenty-four hours after plating, add lentivirus-H2B-mNeonGreen or lentivirus-H2B-mCherry to the cells with a multiplicity of infection (MOI) of 1–10.
  c. 48–72 h after transduction, when the cells reach 70% confluency, passage the cells to two new wells and select the labeled cells with antibiotics, dependent on the resistance gene present in the lentivirus construct. In our experiments, we used 1 μg/mL puromycin to select the H2B-mNeonGreen positive cells and 10 μg/mL blasticidin to select H2B-mCherry positive cells.
    ***Note:*** The concentration of the antibiotic is dependent on the cell line and antibiotic batch. Determine the concentration needed for selection beforehand with an antibiotic kill curve (Sigma Aldrich, 2021).
  d. Expand the selected population in a T25 or T75 flask before the start of the experiment.
    ***Note:*** When transduced cells are cultured for longer periods of time, non-transduced cells can slowly take over and decrease the percentage of fluorescently labeled cells. To avoid this, adding a low concentration of antibiotics to the standard culture medium is recommended. We added 1/5^th^ of the concentration used for the selection of cells to the culture medium for maintenance.

### Preparation of equipment and materials for mouse brain collection and vibratome slicing

**Timing: 30 min**

Right before the start of the experiment, perform the following preparations within a biosafety cabinet level 1:

3. Prepare the culture plates for collection of the *ex vivo* organotypic brain slices.
  a. Prepare 1.5 mL serum-supplemented recovery medium per well for a 6-well plate, by adding 0.375 mL FBS to 1.125 mL recovery medium base per well and transfer to a 6-well plate. The number of wells is dependent on the experimental conditions.
  b. Place cell culture inserts into the serum-supplemented recovery medium using forceps.
  c. Place the plate at 37°C to preheat in a humidified 5% CO_2_ incubator until further use.

***Note:*** Optionally, the *ex vivo* organotypic brain slices can be collected first in cell culture inserts placed in recovery medium without FBS to avoid exposure of the serum-supplemented recovery medium to the open air. In this case, prepare an additional 6-well plate with serum-free recovery medium, by adding 0.375 mL plain DMEM/F-12 medium to 1.125 mL recovery medium base per well.

4. Transfer 100 mL artificial cerebrospinal fluid (aCSF) to a sterile flask and keep it on ice.
5. Transfer 25 mL aCSF to a 50 mL tube for collection of the mouse brain tissue.
6. Clean the vibratome and additional equipment.
  a. Clean the vibratome and working space around it with 70% ethanol.
  b. Clean the buffer tray and place it in the vibratome.
  c. Fill the space around the buffer tray with ice.
  d. Sterilize the vibratome equipment (razor blades, specimen disc, brushes, and spoons) by incubating it in the vibratome buffer tray with a layer of 70% ethanol until further use.

***Optional:*** The vibratome can be placed in a laminar flow cabinet to reduce the risk of contamination. This is however not required when the aseptic techniques specified in step 6 are applied.

## Key resources table

**Table:** 

REAGENT or RESOURCE	SOURCE	IDENTIFIER
**Antibodies**		

Chicken anti-vimentin (dilution 1:1500)	Chemicon	AB5733, RRID: AB_11212377
Rabbit anti-laminin (dilution 1:1000)	Sigma-Aldrich	L9393, RRID: AB_477163
Rat anti-BrdU (dilution 1:500)	Bio-Rad	OBT0030S, RRID: AB_609570
Donkey anti-chicken Alexa Fluor Cy5 (1:1000)	Jackson ImmunoResearch	703-175-155, RRID: AB_2340365
Donkey anti-rabbit Alexa Fluor Cy3 (1:1000)	Jackson ImmunoResearch Laboratories	711-166-152, RRID: AB_2313568
Donkey anti-rabbit Alexa Fluor 647 (1:1000)	Jackson ImmunoResearch Laboratories	711-606-152, RRID: AB_2340625
Donkey anti-rat Alexa Fluor 647 (1:1000)	Jackson ImmunoResearch Laboratories	712-605-153, RRID: AB_2340694

**Chemicals, peptides, and recombinant proteins**		

DMEM/F-12	Gibco	11320033
DMEM/F-12 GlutaMAX^tm^ supplement	Gibco	10565018
DMEM high glucose	Gibco	41966052
F10 nutrient mix	Gibco	22390025
L-Glutamine	Gibco	25030081
Phosphate buffered saline (PBS) 10×	Gibco	14200-067
Penicillin-Streptomycin (10,000 U/mL)	Gibco	15140122
Puromycin Dihydrochloride	Gibco	A1113803
Fetal bovine serum (FBS)	Biowest	S181H
Blasticidin	InvivoGen	ant-bl-1
Recombinant human EGF	PeproTech	AF-100-15-A
Recombinant human FGF-basic	PeproTech	AF-100-18B
Polyethylenimine (PEI)	Polysciences	23966-100
5-Bromo-2′-deoxyridine (BrdU)	Sigma-Aldrich	B5002-1G
HEPES (238.30 g/mol)	Sigma-Aldrich	7365-45-9
Sodium azide (65.01 g/mol)	Sigma-Aldrich	S2002-100G
Saponin	Sigma-Aldrich	47036
D-Glucose (198.17 g/mol)	Sigma-Aldrich	16301-1KG
Boric acid (61.83 g/mol)	Merck KGaA	10043-35-3
NaHCO3 (84.01 g/mol)	Merck KGaA	144-55-8
NaH_2_PO_4_ (137.99 g/mol)	Merck KGaA	10049-21-5
MgCl_2_ (203.30)	Merck KGaA	7791-18-6
CaCl_2_ (147.02)	Merck KGaA	10035-04-8
Glycerol	Merck KGaA	55-81-5
Paraformaldehyde	Merck KGaA	30525-89-4
Sucrose	Merck KGaA	57-50-1
Hydrochloric acid fuming 37%	Merck KGaA	100317
2-Methylbutan	Carl Roth	3927.1
KCL (74.55 g/mol)	VWR Chemicals	7447-40-7
Gelatin	VWR Chemicals	24360.233
Thimerosal	GERBU Biotechnik GmbH	1031.0010
Normal Donkey Serum	Jackson ImmunoResearch	017-000-121, RRID: AB_2337258
Triton X-100	Roche Diagnostics GmbH	9036-19-5
Tissue Tek	Sakura	4583
RapiClear 1.47	SunJin Lab. Co	#RC147001
Hoechst 33528	Thermo Fischer Scientific	H3569
FluorSave^TM^ Reagent	Millipore	345789

**Experimental models: Cell lines**		

U251-MG, 1 to 20 passages after thawing	ECACC	09063001
293T/17, 1 to 20 passages after thawing	ATCC	ACS-4500

**Experimental models: Organisms/strains**		

C57BL6J, postnatal day 15–17, male and female	Charles Rivers Laboratories	Strain code 027

**Recombinant DNA**		

pLenti6-H2B-mCherry	Laboratory of Torsten Wittman	Addgene #89766
pLV-H2B-mNeonGreen	Laboratory of Hugo Snippert (Drost et al., 2015)	N/A
pMD2.G (VSVG)	Laboratory of Didier Trono	Addgene #12259
pMDLg/PRRE	Laboratory of Didier Trono	Addgene #12251
pPRSV rev	Laboratory of Didier Trono	Addgene #12253

**Software and algorithms**		

Fiji (ImageJ version 1.53c)	Schneider et al. (2012)	RRID: SCR_002285, https://imagej.net/software/fiji/downloads
Imaris version 8.4 and version 9.4	Bitplane	RRID: SCR_007370, https://Imaris.oxinst.com/

**Other**		

Allegra 25R Centrifuge	Beckman Coulter	BA-TB-001A
Lenti-X™ GoStix™ Plus	Takara Bio	631281
Falcon® Permeable Support for 6-well Plate with 1.0 μm Transparent PET Membrane, Sterile	Corning	353102
Corning tissue-culture treated culture dishes (15 cm^2^)	Merck	CLS430599
Corning 500 mL Bottle Top Vacuum Filter, 0.22 μm	Merck	CLS431117
Corning 50 mL centrifuge tubes	Merck	CLS430829-500EA
Scott Duran Flask 1 L	VWR	215-1595
Scott Duran Flask 100 mL	VWR	215-1592
Forceps	Fine Science Tools	11080-02
Fine Scissors	Fine Science Tools	14184-09
Surgical Scissors	Fine Science Tools	14101-14
Loctite 401 glue	Henkel Adhesives	123011
Stainless Steel Blade	Campden Instruments Limited	752/1/SS
Surgical Blade	B. Braun	BB510
Hamilton 0.5 μL syringe model 7000.5 KH	Hamilton	86250
Micromanipulator model MM-3	Narishige group	N/A
GJ-8 magnetic stand	NARISHIGE Group	N/A
Iron plate	NARISHIGE Group	N/A
Leica MS5 stereomicroscope	Leica Microsystems	N/A
Horizontal orbital shaker, benchmark incu-shaker mini	Benchmark Scientific Inc, USA	H1001-M
iSpacers (0.5 mm)	SunJin Lab co.	#IS002
Peel-A-way embedding molds	Sigma-Aldrich	E6032
VT1000S Vibratome	Leica Biosystems	1404723512
Buffer tray	Leica Biosystems	14046230132
Specimen disc S D 50 mm	Leica Biosystems	14046327404
Knife holder S	Leica Biosystems	14046230131
Leica CM 1950 cryostat	Leica Biosystems	1491950C
SuperFrost Plus Adhesion microscope slides	VWR	631-0108
LSM 880 confocal microscope with 3-channel QUASAR Detection unit	ZEISS	N/A
N-Achroplan 10×	ZEISS	420940-9901-000
LD Plan-NEOFLUAR 20×	ZEISS	421350-9971-000
AxioVert A1 epifluorescent microscope	ZEISS	N/A
AxioScope A1 epifluorescent microscope	ZEISS	N/A

## Materials and equipment

**Table:** U251-MG cell culture medium

Reagent	Final concentration	Amount
DMEM high glucose	44.5% (v/v)	222.5 mL
F10 nutrient mix	44.5% (v/v)	222.5 mL
Fetal bovine serum	10% (v/v)	50 mL
Penicillin/streptomycin	1.0% (v/v)	5 mL
**Total**	**N/A**	**500 mL**

The medium can be stored for 4 weeks at 4°C.

**Table:** 293T/17 cell culture medium

Reagent	Final concentration	Amount
DMEM high glucose	89% (v/v)	445 mL
Fetal bovine serum	10% (v/v)	50 mL
Penicillin/streptomycin	1.0% (v/v)	5 mL
**Total**	**N/A**	**500 mL**

The medium can be stored for 4 weeks at 4°C.

**Table:** Artificial cerebrospinal fluid (aCSF)

Reagent	Final concentration	Amount
HEPES	10 mM	2.384 g
NaHCO_3_	21 mM	1.76 g
NaH_2_PO_4_	1.2 mM	164 μg
KCl	2.5 mM	185 μg
MgCl_2_	2 mM	410 μg
CaCl_2_	2 mM	294 μg
D-glucose	5 mM	999 μg
Glycerol	250 mM	25 mL
ddH_2_O	N/A	Up to 1 L
**Total**	**N/A**	**1 L**

After dissolving the chemicals in 800 μL ddH2O, measure and adjust the pH and fill up to 1 L. Afterward, filter using a 0.22 μm bottle-top vacuum filter and store the aCSF at 4°C. aCSF can be used until precipitates start to form.

**Table:** Recovery medium base (1.33×)

Reagent	Final concentration	Amount
HEPES	6.65 mM	0.396 g
NaHCO_3_	0.9% (w/v)	2.35 g
Penicillin/streptomycin	1.33% (v/v)	3.33 mL
DMEM/F-12	N/A	246.67 mL
**Total**	**N/A**	**250 mL**

Dissolve the HEPES and NaHCO_3_ in 185 mL DMEM/F-12, filter through a 0.22 μm bottle-top vacuum filter. Add penicillin/streptomycin and store at 4°C, can be stored for 4 weeks.

**Table:** Serum-supplemented recovery medium

Reagent	Final concentration	Amount
FBS	25%	2.5 mL
Recovery medium base	75%	7.5 mL
**Total**	**N/A**	**10 mL**

Volume specified is for one 6-wells plate. Prepare fresh before the start of the experiment.

**Table:** Serum-free recovery medium

Reagent	Final concentration	Amount
DMEM/F-12	25%	2.5 mL
Recovery medium base	75%	7.5 mL
**Total**	**N/A**	**10 mL**

Volume specified is for one 6-wells plate. Prepare fresh before the start of the experiment.

**Table:** Neural stem cell (NSC) medium

Reagent	Final concentration	Amount
DMEM/F-12 with GlutaMAX^tm^	N/A	14.82 mL
Penicillin/streptomycin	1% (v/v)	150 μL
EGF (10 μg/mL)	10 ng/mL	15 μL
FGF (10 μg/mL)	10 ng/mL	15 μL
**Total**	**N/A**	**15 mL**

After adding the EGF and FGF, the medium should be stored at 4°C and be used within a week. Adjust the volume to the amount needed for a 1-week experiment.

***Note:*** In the medium of the original protocol, 2% StemPro supplement is added to the medium (Pencheva et al., 2017). Although we used the medium without this component, it can be added to the medium when problems with tissue survival are experienced.

**Table:** PBS-Gelatin-Triton X-100 (PBS-GT) buffer

Reagent	Final concentration	Amount
Gelatin	0.2% (w/v)	0.5 g
Triton-X-100	0.5% (v/v)	1.25 mL
Thimerosal	0.01% (w/v)	25 μg
PBS (10×, pH 7.4)	1×	25 mL
ddH_2_O	N/A	up to 250 mL
**Total**	**N/A**	**250 mL**

Buffer can be stored for 2 weeks at 4°C.

**CRITICAL:** Thimerosal contains mercury and should be handled with extra caution. Solutions containing this compound should be handled in a fume hood. Thimerosal can be replaced by sodium azide (0.01%), this chemical is also toxic and should be handled in a fume hood.

**Table:** 4% PFA in PBS (pH 7.4)

Reagent	Final concentration	Amount
Paraformaldehyde	4% (w/v)	4 g
PBS (10×, pH 7.4)	1×	10 mL
ddH_2_O	N/A	up to 100 mL
**Total**	**N/A**	**100 mL**

Dissolve the paraformaldehyde in 50 mL ddH_2_O by heating and stirring the solution up to 60°C (do not boil) and by adding 5 N NaOH drop by drop until a clear solution is formed. Add 10× PBS, let the solution cool down, adjust the pH to 7.4 and adjust the volume to 100 mL with ddH2O. Filter the solution, aliquot and freeze the aliquots at −20°C for long-term storage. Avoid repeated thaw-freeze cycles.

**Table:** Blocking buffer

Reagent	Final concentration	Amount
10% Triton-X-100 in PBS	0.5% (v/v)	0.5 mL
Normal Donkey Serum	5.0% (v/v)	0.5 mL
PBS (10×, pH 7.4)	1×	1 mL
ddH_2_O	N/A	8 mL
**Total**	**N/A**	**10 mL**

Store at 4°C for 1 week or −20°C for long-term storage.

**Table:** 2 N HCL

Reagent	Final concentration	Amount
Hydrochloric acid (37%)	2 mol/L	16.6 mL
ddH_2_O	N/A	83.4 mL
**Total**	**N/A**	**250 mL**

Solution is stable at room temperature (19°C–21°C).

**Table:** 0.1 M Borate Buffer (pH 8.5)

Reagent	Final concentration	Amount
Boric acid	0.1 M	6.18 g
ddH_2_O	N/A	up to 100 mL
**Total**	**N/A**	**1 L**

Dissolve in 800 mL and adjust the pH to 8.5. The solution is stable at room temperature (19°C–21°C).

### Microscopy

• LSM 880 confocal microscope.
  ○ 3-channel QUASAR Detection unit.
  ○ N-Achroplan 10×.
  ○ LD Plan-NEOFLUAR 20×.

## Step-by-step method details

### Dissection of postnatal mouse brains (day 1)

**Timing: 30 min**

This section describes the dissection of a postnatal mouse brain that will be used for the preparation of *ex vivo* organotypic brain slices later in the protocol.

1. Sacrifice a 16 days-old C57BL/6 mouse by exposure to CO_2_ and decapitate using scissors.

***Note:*** We selected the mouse breed and timepoint based on earlier reports in literature (Markovic et al., 2005; Sliwa et al., 2007). However, older animals and different breeds have also been successfully used to make brain slices (Pencheva et al., 2017). Please refer to Humpel, 2015 for further explanation on the different ages and (dis)advantages.

***Note:*** The use of cervical dislocation or guillotine is not recommended, as it can damage the brain tissue before dissection.

2. Peel away the skin and meninges of the head until the skull becomes visible (Figures 1A-I,II).

**Figure 1:**
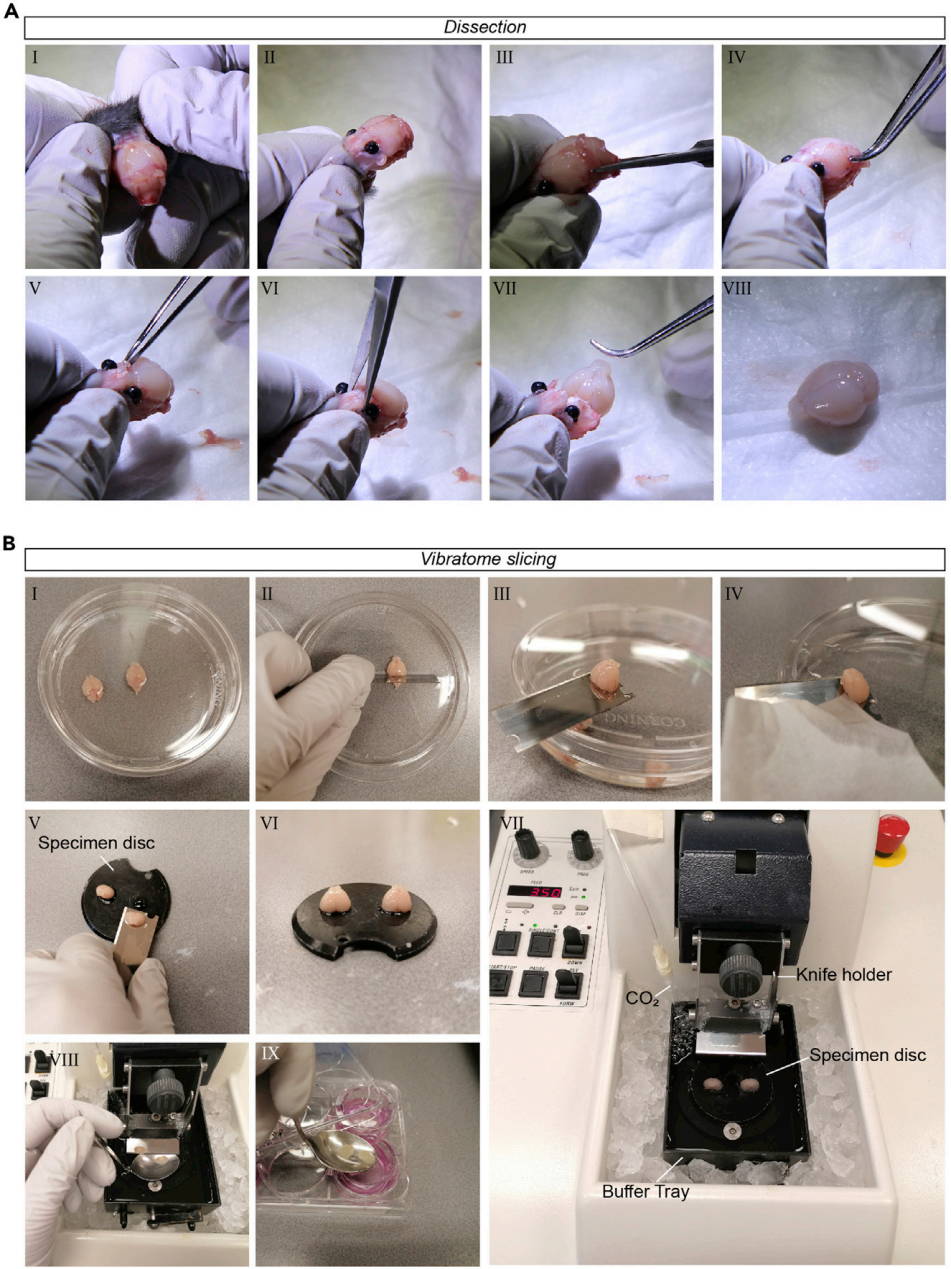
Mouse brain dissection and vibratome slicing of *ex vivo* organotypic brain slices (A) Example images of dissection steps described in step 2 (I, II), step 3 (III), step 4 (IV, V), step 5 (VI) and step 6 (VII, VIII) of the step-by-step method details. Pictures were taken with a digital photography camera. (B) Example images of vibratome slicing steps described in step 9 (I, II), step 10 (III, IV), step 11 (V,VI), step 14 (VII), step 17 (VIII, IX) of the Step-by-Step Methods Details. Pictures were taken with a smartphone camera.
3. Place ultra-fine scissors in the spinal cord and cut until the cerebellum is reached (Figure 1A-III).
4. Carefully peel away the skull meninges using forceps until the brain is uncovered (Figures 1A-IV, V).
5. With ultra-fine scissors make a cut rostral of the olfactory bulb to disconnect the olfactory nerves (Figure 1A-VI).
6. Place forceps caudally under the brain and transfer the brain from the skull into a 50 mL tube containing 25 mL aCSF and place it on ice (Figures 1A-VII,VIII).
7. Repeat the procedure for the other mice when multiple brains are needed.

### Preparation of *ex vivo* organotypic brain slices using the vibratome (day 1)

**Timing: 45 min**

In these steps, we describe the procedure of making brain slices from freshly dissected mouse brains using the vibratome. The procedure described below is adapted from Pencheva et al. (2017).

8. Take the vibratome equipment out of the buffer tray with 70% ethanol, remove the ethanol and let everything air-dry.
9. Pour the aCSF with the brain(s) into a sterile Petri dish (Figure 1B-I) and remove the olfactory bulb and cerebellum using a sterile razor blade (Figure 1B-II).
10. Place the brain on the razor blade with the rostral part facing upward (Figure 1B-III) and remove residual aCSF from the caudal part by lightly touching the brain-blade interface with a paper tissue (Figure 1B-IV).
11. Place a drop of Loctite 401 instant adhesive glue on the specimen disk and transfer the brain to the glue with the rostral part facing up (Figures 1B-V,VI).

***Note:*** Two additional brains can be transferred to the same specimen disk to slice three brains simultaneously. In this case, make sure that all brains are aligned and oriented in the same direction.

12. Place the specimen disc with the mounted brain tissue into the buffer tray and fixate.
13. Fill the buffer tray with cold aCSF until the tissue is fully submerged (100 mL).
14. Place a filter tip connected to CO_2_ into the bucket to carbonate the aCSF (Figure 1B-VII).
15. Turn on the vibratome, set the frequency to 7 Hz and the slicing thickness to 350 μm.
16. Start trimming off the rostral region of the brain and stop when the lateral ventricles become visible.
17. Take the culture plate with the cell inserts out of the incubator and start collecting tissue with visible lateral ventricles. To collect the tissue:
  a. Place a sterile spoon underneath the blade and carefully transfer the tissue to the spoon using a sterile brush (Figure 1B-VIII).
  b. Pour off most of the aCSF and transfer the brain slice to a cell culture insert (Figure 1B-IX).
18. After collecting four slices per insert, remove the residual aCSF using a P1000 pipette with filter tip and take the plate to a cell culture laminar flow cabinet.
19. Wash the slices once with sterile PBS.

***Optional:*** When the slices are collected in serum-free recovery medium, transfer the cell culture inserts to the culture plate with serum-supplemented recovery medium after the PBS wash.

20. Place the plate with cell culture inserts containing the brain slices in serum-supplemented recovery medium into a 37°C with 5% CO_2_ incubator for recovery of the brain slices.
21. Culture the brain slices overnight (min 24 h).

### Injection of glioma cell lines into the *ex vivo* organotypic brain slices (day 2)

**Timing: 2–4 h**

This section describes the preparation of glioma cell suspensions and the injection of the cells into the *ex vivo* organotypic brain slices (Methods video S1). The timing needed for these steps is dependent on the number of cell lines and the number of brain slices that will be used in the experiment.

22. Prepare the cell-suspensions:
  a. Passage the cells used for the experiment with standard passaging methods.
  b. Make cell suspensions of 2.5 × 10^4^ cells/ μL. A minimal volume of 5 μL (1.25 × 10^5^ cells) is needed.

***Optional:*** The invasive and proliferative properties of the experimental group can be directly compared to an internal control group. When choosing for this experimental set-up, mix cells of the internal control cells with fluorophore A one to one with the experimental group expressing fluorophore B. We used H2B-mCherry expressing control cells as an internal control, in combination with experimental groups expressing H2B-mNeonGreen.

23. Transfer the *ex vivo* organotypic brain slices:
  a. Prepare a 6-well plate with 1 mL neural stem cell (NSC)-medium per insert.
  b. Prepare a 6-well plate with 2 wells with 1 mL PBS per insert.
  c. Take the cell culture inserts with *ex vivo* organotypic brain slices from the incubator.
  d. Dip the inserts two times in 1 mL PBS.
  e. Remove the remaining recovery medium from the inserts and transfer the inserts to the 6-well plate with NSC-medium.
24. Prepare the micromanipulator set-up (Figure 2A-I):
  a. Clean your working space with 70% ethanol.
  b. Fixate the micromanipulator at the dissection microscope using the magnetic stand.
  c. Place the Hamilton syringe in the micromanipulator.
  d. Clean the syringe by washing it first with acetone (1×) and, subsequently, multiple times with 70% ethanol. Finally, rinse the syringe with PBS.

**Figure 2:**
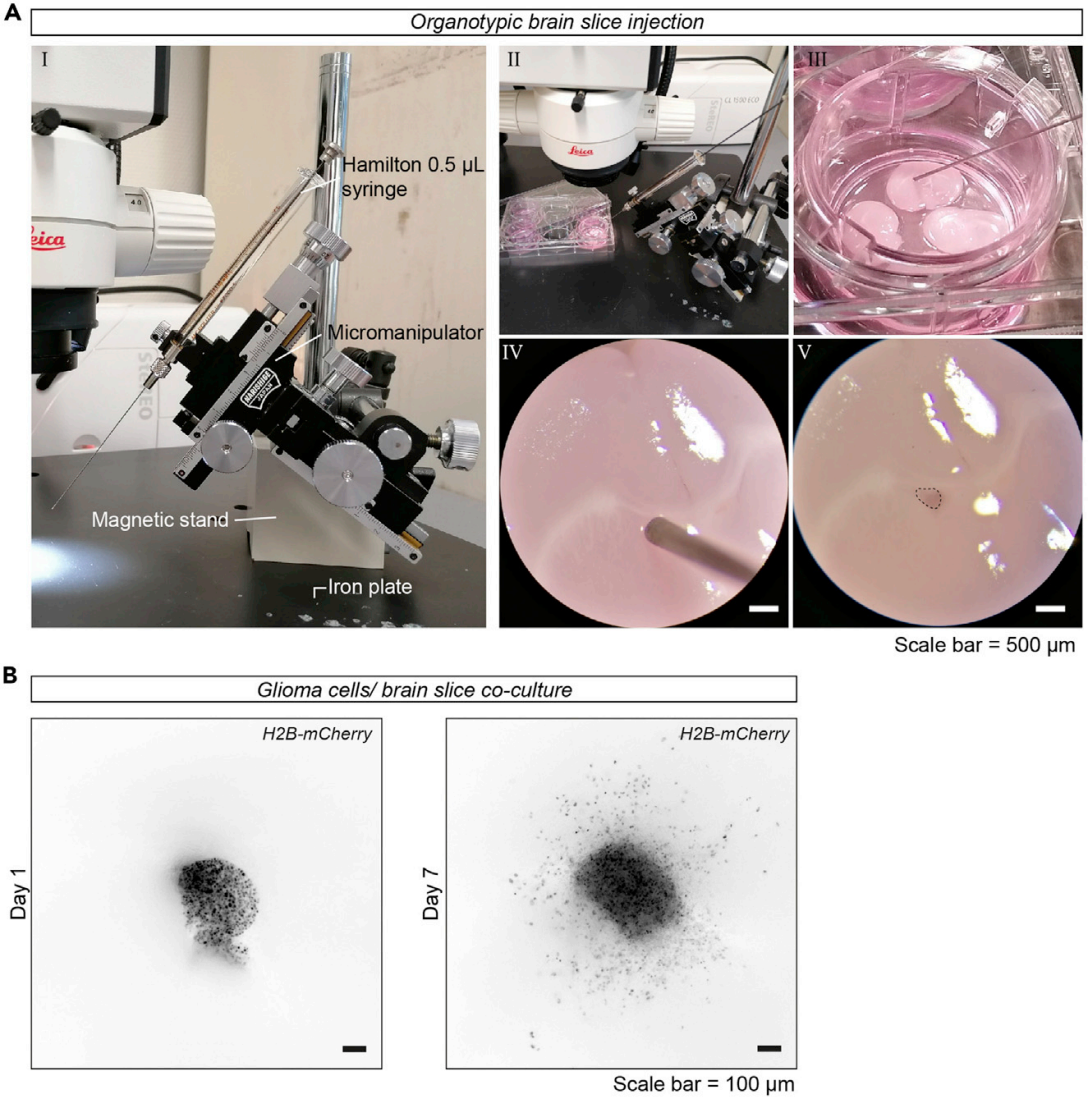
Injection and co-culture of glioma cells in *ex vivo* organotypic brain slices (A) Injection of slices using a micromanipulator. (I) Image of the micromanipulator set-up, with an iron plate, a magnetic stand, a micromanipulator, and a Hamilton syringe. (II, III, IV) Example images of an organotypic brain slice in a cell-culture insert with a Hamilton syringe inserted into the lateral ventricle of the brain slice. (IV, V) Example images of the organotypic brain slice observed through a dissection microscope before (IV) and after (V) injection with the glioma cells. The dotted line indicates the location of the injected cells. Scale bar = 500 μm. Pictures were taken with a smartphone camera (IV and V through a Leica MS5 stereomicroscope). (B) Epifluorescent images of injected H2B-mCherry expressing U251-MG cells on day 1 (I) and day 7 (II) in culture. Cells in focus are migrating at the membrane/ brain slice interface. Scale bar = 100 μm. Images are taken with an AxioVert A1 epifluorescent microscope.
25. Orient the plate with organotypic brain slices and the micromanipulator under the dissection microscope.
  a. Place the 6-well plate with the organotypic brain slices under the dissection microscope and remove the lid.
  b. Move the micromanipulator and orient in such a way that the syringe is in the center of view at 4× magnification when touching the organotypic brain slices (Figures 2A-II,III,IV).
26. Inject the *ex vivo* organotypic brain slices with the glioma cell suspensions prepared in step 22:
  a. Mix the cell suspension with a normal pipette and directly take up 0.5 μL with the syringe.
  b. Using the micromanipulator, move the syringe to the brain slice. Slowly insert the syringe ∼ 50 μm into the lateral ventricle at an angle of approximately 45 ˚ and afterwards retract it 40 μm out of the tissue.
  c. Inject the cell suspension into the lateral ventricle at low speed (∼30 s per 0.5 μL), to prevent the cells from flushing over the tissue (Figure 2A-V, Methods video S1).
  d. Slowly take out the syringe after injection.
  e. Rinse the syringe with PBS multiple times before injecting the next cell line.
27. After finishing all the injections, rinse the syringe with PBS, 70% ethanol and acetone.
28. Inspect the organotypic brain slices with a fluorescent microscope to check the presence of fluorescently labeled cells (Figure 2B).
29. Place the culture plate in the incubator to culture the *ex vivo* organotypic brain slices.

Methods video S1. Injection of glioma cells into *ex vivo* organotypic brain slices, related to step 26

### Culturing and fixation of the *ex vivo* organotypic brain slices (day 2 – day 8)

**Timing: 7days**

Below we describe the procedure for culturing the injected organotypic brain slices and fixating them at the end of the co-culture period.

30. Replace the medium of the injected organotypic brain slices once every 2 or 3 days by transferring the inserts into a new well with 1 mL of fresh prewarmed NSC-medium.
31. At day 8 (on the 7^th^ day after injection of cells), fixate the *ex vivo* organotypic brain slices.
  a. Wash the cell culture inserts containing the brain slices one time with PBS.
  b. Place the inserts in 2 mL 4% PFA in PBS (covered from light) at 4°C overnight (min 12 h).
32. The next day, wash the inserts 3 times with PBS and store at 4°C until further use.

***Note:*** The migratory patterns of the glioma cells can be followed during the culturing period using epifluorescence microscopy (Figure 2B). Note, however, that the cells that can be observed with an inverted microscope are the cells that migrate on the membrane rather than into the tissue. To visualize and analyze the cells migrating into the tissue, follow the steps described below.

**Pause point:** The fixated organotypic brain slices can be stored in PBS for multiple weeks before further processing. Anti-fungal agents can be added when the slices are not used within a month.

### Whole-mount immunostaining and tissue clearing

**Timing: 6days**

In this section, we describe a whole-mount co-immunostaining protocol for the extracellular matrix (ECM) protein laminin and the intermediate filament protein vimentin and describe clearing- and tissue mounting steps required for imaging of the samples. Immunostaining for laminin labels both the vasculature of the mouse brain tissue, as well as the ECM proteins produced by the injected glioma cells at the injection site. The latter allows for distinction between the tumor core and invasive front used during later analysis steps. Vimentin can be used to visualize the larger cellular processes of the glioma cells. The described protocol is an adaptation of the whole-mount immunostaining protocol developed by (Belle et al., 2014) and the RapiClear 1.47 clearing method developed by the SunJin Lab (www.sunjinlab.com, n.d.; Bekkouche et al., 2020).

33. Cut the membrane from the inserts around the individual brain slices using a scalpel and transfer the membrane with the brain slice attached to it to a 24-well plate.
34. Permeabilize the brain slices in 500 μL 2% PBST (2% Triton-X100 in PBS) at room temperature (19°C–21°C) for 4 h.
35. Add 500 μL PBS-GT (materials and equipment) to the brain slices and incubate at room temperature (19°C–21°C) overnight (min 12 h).

**CRITICAL:** The PBS-GT contains thimerosal (a mercury solution) and should be handled with caution, handle solutions containing this compound in a fume hood.

36. Incubate the brain slices with primary antibodies:
  a. Add primary antibodies to PBS-GT with 0.1% saponin, prepare 250 μL primary antibody mix per sample.
  b. Add the mix to the brain slices and incubate at 37°C on a horizontal shaker (70 rpm) for 72 h. Here we used rabbit anti-laminin (1:1 000, specific for human, mouse, and other species) and chicken anti-vimentin (1:1 500, specific for human, mouse, and other species) antibodies.
37. Wash the brain slices 6 times with PBS-T (0.5% Triton-X100 in PBS) for 1 h per wash (total of 6 h) at room temperature (19°C–21°C).
38. Incubate the brain slices with secondary antibodies:
  a. Add secondary antibodies to PBS-GT with 0.1% saponin, prepare 250 μL primary antibody mix per sample.
  b. Add to the brain slices and incubate at 37°C on a horizontal shaker (70 rpm) for 24 h. Protect from light by covering the well plate with aluminum foil. Here we used donkey anti-rabbit-Cy3 (1:1 000) and donkey anti-chicken-AF647 (1: 1 000) secondary antibodies.
39. Wash the brain slices 6 times with PBS-T at room temperature (19°C–21°C) for 1 h per wash step (total of 6 h).
40. Incubate the brain slices with clearing solution:
  a. Pre-warm RapiClear 1.47 solution at 37°C and add 300 μL to each brain slice.
  b. Place on a horizontal shaker at 37°C and incubate for a minimum of 45 min until the brain slice is transparent.

***Note:*** The used RapiClear solution can be reused maximally three times.

**Figure 3:**
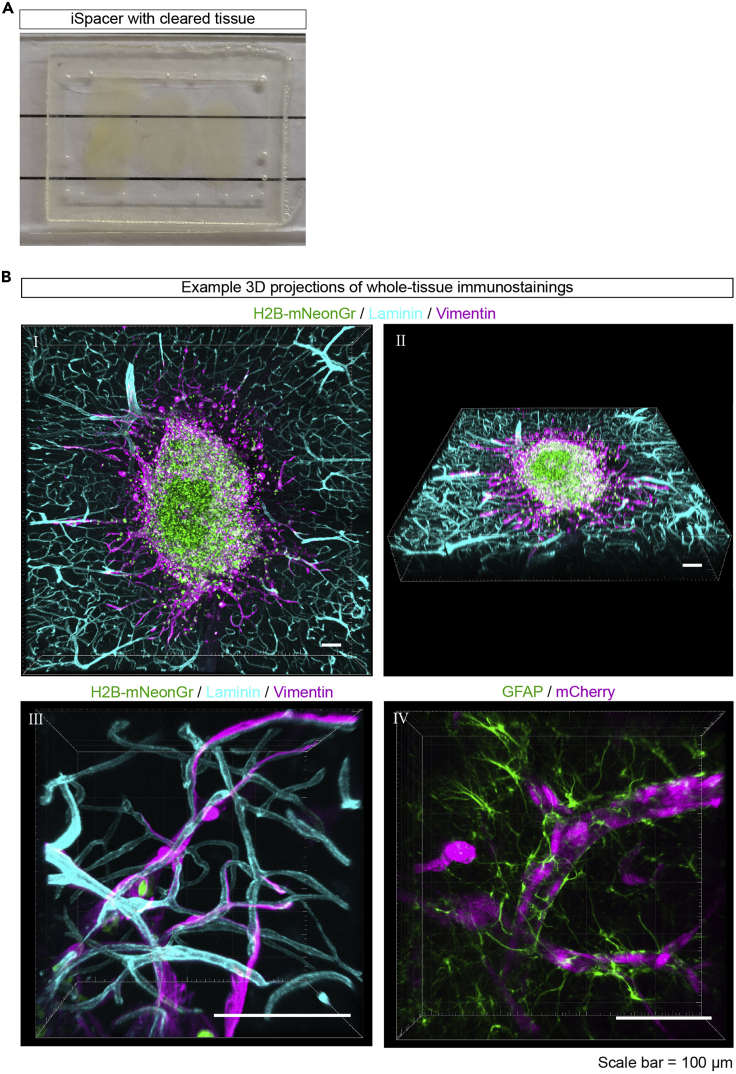
Whole-mount immunostaining and tissue clearing of *ex vivo* organotypic brain slices (A) Image of four brain slices placed within an iSpacer on a microscope glass slide, covered and cleared with RapiClear, and mounted with a coverslip. Pictures were taken with a smartphone camera. (B) Example 3D projection images of whole-mount immunostainings of brain slices injected with H2B-mNeonGreen (green) expressing U251-MG cells. (I, II) Visualization of the mouse brain vasculature with laminin (cyan) and the glioma-cell cytoskeleton with vimentin (magenta). (III) A higher magnification image of individual glioma cells migrating along the mouse brain vasculature. (VI) Visualization of mouse brain astrocytes with GFAP (green) and invading mCherry expressing U251-MG cells (magenta). Scale bar = 100 μm. Images were taken with a LSM 880 confocal microscope.
  b. Transfer the brain slices to the microscopy slide. Multiple brain slices can be mounted on one glass microscopy slide.
  c. Fill the iSpacer with fresh RapiClear solution and seal with a coverslip.
  d. Use clear nail polish to seal the edges of the coverslip.
42. Store the microscopy slides at 4°C until further use.

***Note:*** The membrane can become detached during the procedure. In this case, use a spatula to transfer the brain to the iSpacer. If the brain slice is still attached, use forceps to transfer the membrane with the brain slice to the iSpacer and orient it in such a way that the membrane is on the bottom of the glass microscopy slide. Orienting it the other way around (i.e., when the membrane is facing the coverslip) may interfere with confocal imaging.

41. Mount the brain slices within iSpacers on a glass microscopy slide:
  a. Place an iSpacer (Figure 3A) on a glass microscopy slide.

### Confocal imaging and image processing

**Timing: 1 h/ brain slice**

This section describes the procedure for confocal imaging of the whole-mount immunostained brain slice and the preparation of the images for image analysis. We used a 10× objective to capture all tumor cells and to create a 3D reconstruction at the macroscopic level. Higher magnification can be used when smaller parts of the tumors are imaged and higher resolution is needed.

43. Place the microscopy slide with the cleared brain slices on the stage of a confocal microscope with the coverslip facing the 10× objective. Navigate to the region of interest.
44. Adjust the laser power and gain to optimize the signal based on background and fluorescent signal.
45. Adjust the number and size of tiles and the top and bottom of the z-stack in such a way that all cells are captured.

***Note:*** Make sure that the pixel size and step size remain constant when imaging different samples within your experiment. For the 10× objective, we used a pixel size of 1.77 μm/pixel and a step size of 6.07 μm as a minimal XY and Z resolution to allow quantification.

46. Stitch the tiled images using the confocal software or using the ‘Stitching of 3D images’ plugin (Preibisch et al., 2009) in ImageJ. We used four tiled images of 576 × 576 pixels each to capture the tumor region.

### Annotation and selection of the tumor core based on laminin deposits using ImageJ

**Timing: 15 min/ brain slice**

Here we describe how you can use the laminin immunostaining to distinguish glioma cells residing in the tumor core from those invading the tissue. We explain how to use ImageJ to create an additional channel in the image file where only the laminin signal associated with the tumor core is selected (see also Methods video S2).

***Optional:*** Before the start of the analysis, use a filename randomizer plugin to blind the images when comparing different experimental conditions.

47. Open the confocal-generated and tiled image in ImageJ. This file should consist of minimally two channels: a channel with the nuclei and a channel with laminin staining.
48. Create a selection of the laminin signal around the tumor core (laminin deposits):
  a. Go to a z-plane where the nuclei are clearly visible.
  b. Open the Brightness & Contrast (Image > Adjust > Brightness & Contrast).
    i Linearly increase the laminin signal by sliding the bar above ‘Maximum’ to the left.
    ii Stop adjusting the histogram until the laminin signal in and around the nuclei become clearly visible (= laminin deposit). The laminin signal from the blood vessels will be saturated at this point.
  c. Duplicate the channel with the laminin staining (Image > Duplicate). Duplicate the hyperstack for the laminin channel only, for all slices.
  d. Open the ROI manager (Analyze> Tools> ROI manager).
  e. Go to the first plane in the z-stack.
  f. Draw a region of interest (ROI) around laminin signal of the tumor core.
    i. Select polygon selection tool.
    ii. Determine the border of the tumor core based on the laminin deposit and the density of the nuclei (Figures 4A-I–III).

**Figure 4:**
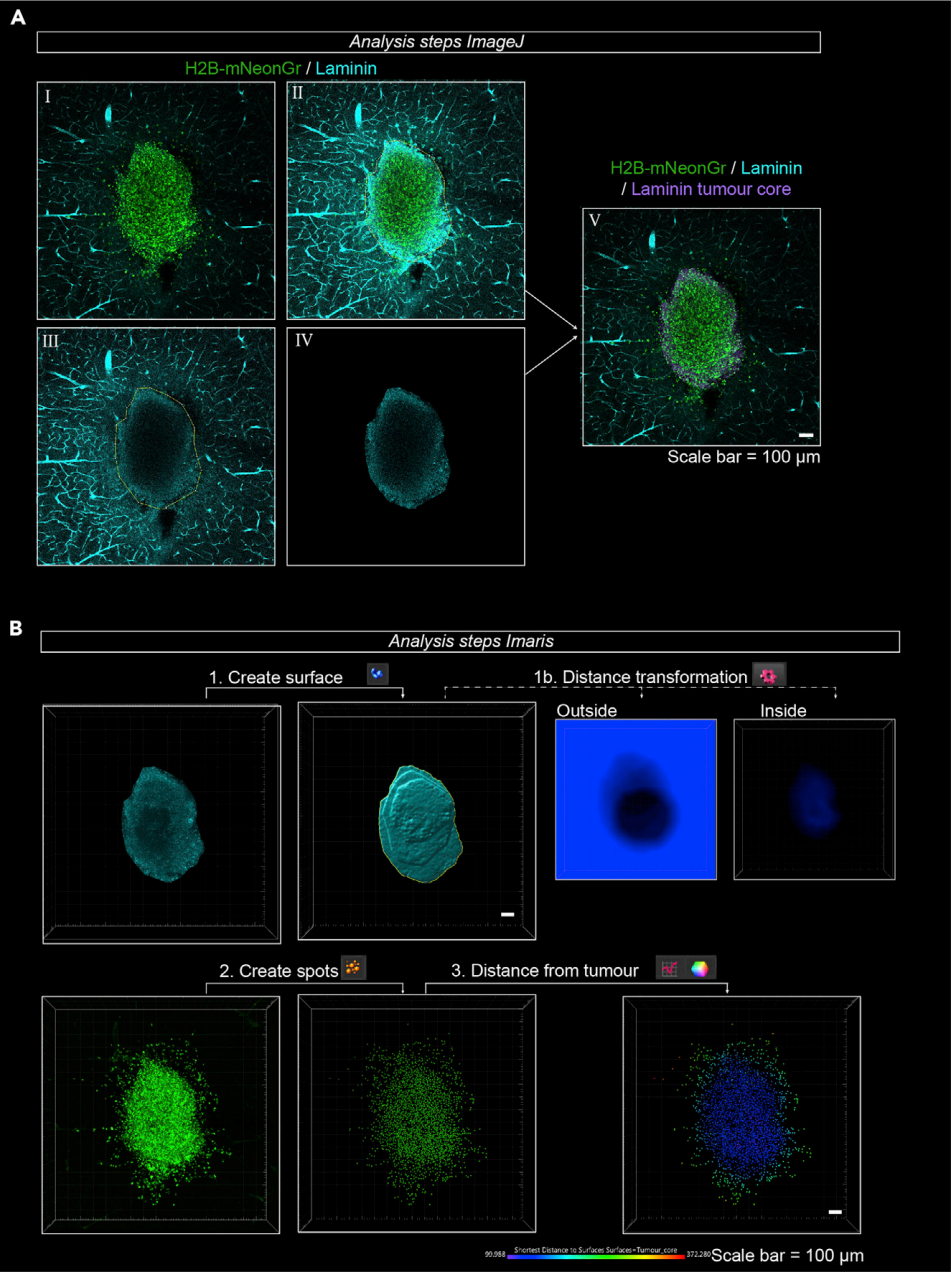
Image processing and analysis steps to quantify invasion of glioma cells (A) Analysis steps in ImageJ for the creation of a laminin tumor core. (I–III) Example of a creation of a ROI around the laminin deposits in the tumor core as described in step 48 of the Step-by-Step Methods Details. (IV) Example of laminin deposit after running the macro to remove the laminin signal not specific to the tumor core described in step 49 of the Step-by-Step Methods Details. (V) Example of the merged image where the laminin core and original file are combined as described in step 50 of the Step-by-Step Methods Details. Scale bar = 100 μm. (B) Analysis steps in Imaris to quantify invasion. Open the file created in ImageJ in the Imaris software. With the ‘Create Surface’ tool, create a surface of the tumor core (1). In Imaris version 9.5 or older, use the Distance transformation option to create new channels where the signal intensity represents the distance to or in the tumor core (1b). In newer versions, this step can be replaced by selecting the option ‘Shortest Distance Calculation’ in the first step of creating a surface. Create spots of the H2B-mNeonGreen channel with the ‘Create Spots’ tool (2). In the newly created spots, the software can calculate, display (3), and export the distance to the surface created in (1). Scale bar = 100 μm.
    iii. Add the selection to the ROI manager by clicking ‘Add[t]’ in the ROI manager.
      ***Note:*** In case no tumor core is visible, in the ROI manager go to ‘More>Specify. In this panel, type the value 1 in ‘Width’, ‘Height’, ‘X coordinate’ and ‘Y coordinate’ and leave the ‘Slice’ value at the plane of interest. After clicking OK, a selection of 1 by 1 pixel will be added to the ROI manager. This step is important for further processing at step 49.
  g. Repeat step 48f for all other z-planes.
  h. Save the ROIs in a designated folder (ROI manager > More> Save).
49. Create an additional channel of the laminin deposits:
  a. Open the ‘Macro’ tool of ImageJ (Plugins > Macros > Startup macros).
  b. Open a new file in the ‘Macros’ tool (File > New). Select the Macro language (Language > IJ1 Macro).
  c. Copy and paste the following script into the ‘Macros’ tool:
    numROIs = roiManager(“count”);
    for(i=0;i<numROIs;i++){
    roiManager(“Select",i);
    run(“Clear Outside","Slice");
    }
  d. Select the duplicated laminin channel and click on ‘Run’ in the Macros tool.
    ***Note:*** After running the macro, the laminin signal outside of the tumor core should have been deleted in every plane and you should be left with the laminin tumor core (Figure 4A-IV).

50. Merge the laminin deposits channel with the original file.
  a. Select the original file.
  b. Split the original file into separate channels (Image > Color > Split Channels).
  c. Merge the separate channels of the original file with the laminin tumor core channel created in step 49 (Image > Color > Merge Channels).
  d. Change the color of the laminin tumor core to distinguish it from the original laminin channel (Figure 4A-V).
51. Save the newly created file (File > Save As).

Methods video S2. ImageJ analysis steps to create a selection of the tumor core based on laminin signal, related to steps 47–51

### Analysis of the distribution of nuclei in tumor core and invasive front using Imaris software

**Timing: 10 min/ brain slice**

This section describes the final analysis steps to quantify the number of invaded and non-invaded glioma cells and to quantify the invasion distances (See also Methods video S3). The analysis steps differ depending on the version of Imaris that is used (versions 8.4–9.4 or version 9.5 or higher). Both versions are described below.

52. Import the files created and saved in step 51 into the Imaris Software and open the first file in the ‘Surpass’ environment.
53. Create a surface (Figure 4B, step 1):
  a. In Imaris version 9.5 or higher, the first panel in the new surface option is ‘Algorithm Settings’. In this panel select ‘Shortest Distance Calculation’.
  b. Select the source channel corresponding to the laminin tumor core. Select the smooth function. In our experiments, we chose a ‘Surface Detail’ of 5 μm.
  c. In the next panel, set the threshold based on ‘Absolute Intensity’. In our experiments, we set the threshold at 2.00.
  d. In the next panel, additional filters can be applied to the created surface. This step can be skipped.
54. Create spots of the individual nuclei in the image (Figure 4B, step 2a):
  a. In Imaris version 9.5 or higher, the first panel in the new surface option is ‘Algorithm Settings’. In this panel select ‘Shortest Distance Calculation’.
  b. Select the source panel corresponding to the nuclei of the injected glioma cells. For spot detection in our experiments, we chose an ‘Estimated XY Diameter’ of 11 μm.
  c. Select ‘Model PSF-elongation along Z-axis’. In our experiments we choose an ‘Estimated Z Diameter’ of 22 μm.
  d. Select ‘Background subtraction’.
  e. In the next panel, set the threshold for the creation of a spot based on the quality of the signal. In our experiments we choose a quality threshold of 10.
    ***Note:*** This step will affect the number of spots that are created and therefore the outcome of your measurements. It is important to optimize these settings.
  f. Finish the creation of the spots by clicking on the green arrows.
  g. Inspect the spots that are created. Spots that are created based on background signal can be manually deleted in the ‘Edit’ panel.
55. *Imaris version 9.5 or higher:* calculate the distance from the individual spots (nuclei) to the created surface (laminin tumor core, Figure 4B, step 3).
  a. Click on ‘Statistics ’and go to ‘Detailed’ and select ‘Specific Values’.
  b. In the dropdown menu, select ‘Shortest Distance to Surfaces Surfaces = x’ in which x corresponds to the name of surface of the laminin tumor core.
  c. Click on the ‘Save’ Icon to export the list as an excel file.
  d. Visualize the distribution of nuclei:
    i. click on the ‘Color’ icon.
    ii. For ‘Color Type’, select ‘Statistics Coded’.
    iii. In the dropdown menu select ‘Shortest Distance to Surfaces Surfaces = x’.

***Note:*** The values and color coding correspond to the closest distance between the individual spots and the border of the surface, with positive values representing nuclei outside of the surface (invaded into the tissue) and negative values representing nuclei within the surface (within the tumor core).

**CRITICAL:** The function ‘Shortest Distance Calculation’ during the first step of creating a new surface (step 53a) or spots (step 54a) is a new feature in Imaris, only present in version 9.5 or higher. For older versions, skip step 55 and continue with step 56.

56. *Imaris version 8.4 – 9.4:* Perform distance transformation on the created surface (Figure 4B, step 1b).
  a. Select the created surface and click on ‘Tools’.
  b. Select the second option: ‘Distance Transformation’. This will start a MATLAB Script embedded within the Imaris Software. Select ‘Outside SurfaceObject’.
  c. Repeat the ‘Distance Transformation’, this time select ‘Inside SurfaceObject’.

***Note:*** After completion of these steps, two new channels will be added. In the first one created in step 56b, the intensity of the signal corresponds to the distance to the outside of the surface. In the second one created in step 56c, the intensity of the signal corresponds to the distance to the surface border within the surface.

57. *Imaris version 8.4 – 9.4:* Calculate the distance from the individual spots (nuclei) to the created surface (laminin tumor core, Figure 4B, step 3).
  a. Select the created spots, click on ‘Statistics’ and go to ‘Detailed’.
  b. In the second dropdown menu, select ‘Intensity Center Ch = x Img 1’, in which Ch = x corresponds to the channel that was created during step 56b (Outside SurfaceObject).
  c. Click on the ‘Save’ Icon to export the data as an excel file.
  d. Repeat the ‘Intensity Center Ch= x Img 1’ step for the channel created during step 56c (Inside SurfaceObject).
  e. Click on the ‘Save’ Icon to export the data as an excel file.
  f. Visualize the distribution of nuclei.
    i. click on the ‘Color’ icon.
    ii. For ‘Color Type’, select ‘Statistics Coded’.
    iii. In the dropdown menu select ‘Intensity Center Ch= x Img 1’ for the channel created during steps 56b or 56c.

***Note:*** The displayed values in steps 57b and 57d correspond to the closest distance of individual spots to the border of the laminin tumor core surface. In the file exported in step 57c, values of 0 correspond to nuclei located within the tumor core, whereas values higher than 0 correspond to nuclei outside the tumor core. In the file exported in step 57e, values of 0 correspond to nuclei located outside the tumor core and values higher than 0 to those inside.

**Figure 5:**
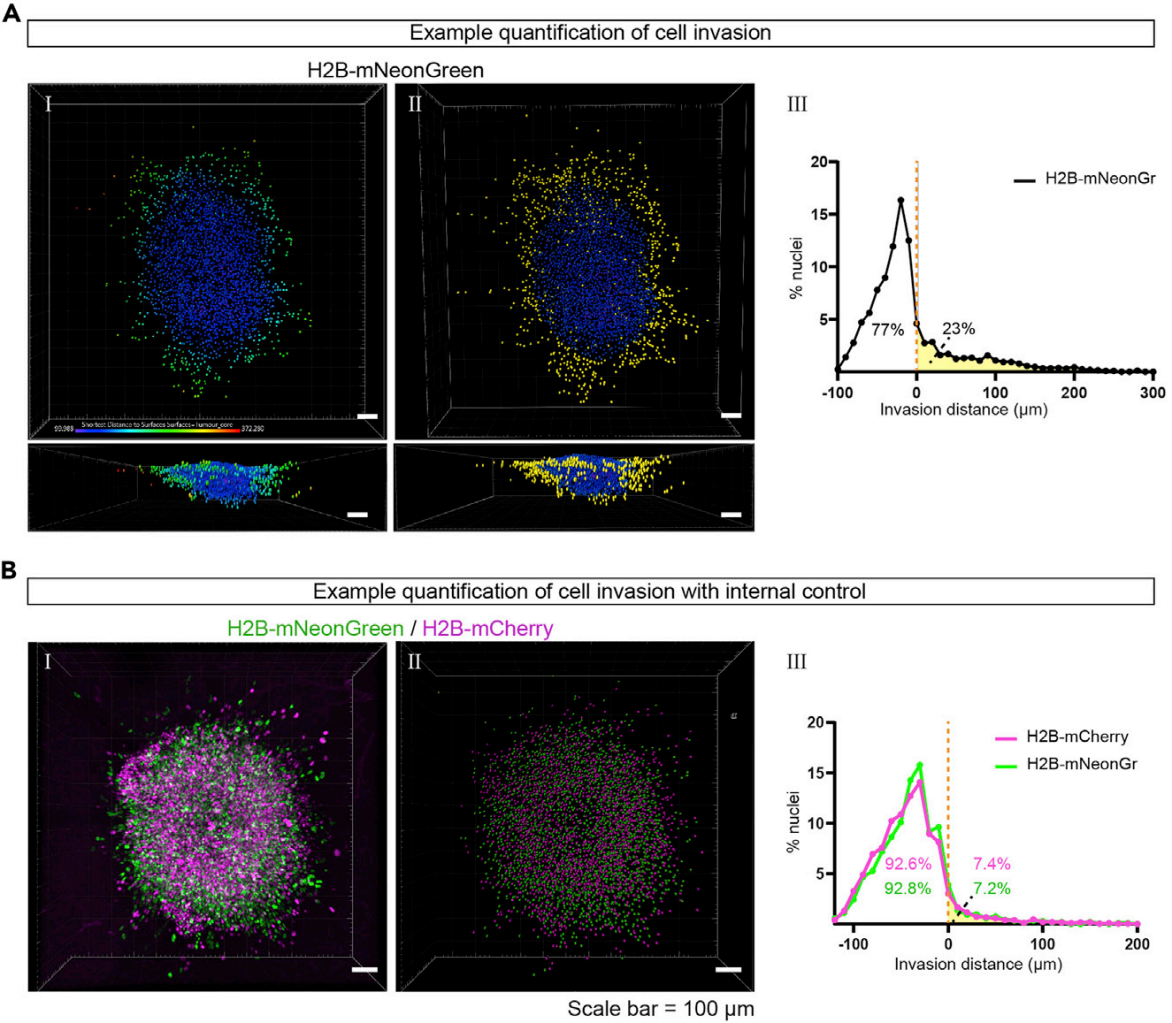
Quantification of cell invasion (A) Example quantification of cell invasion. (I) 3D projection of individual nuclei where every nucleus is color coded based on the closest distance to the border of the tumor core. (II) Selection of nuclei that have a value larger than 0 μm, depicted in yellow, representing the invading population of cells. (III) Histogram of invasion distance represents the distribution of cells in the tumor core (AUC = white) and tissue (AUC = yellow) of the 3D representation displayed in (I). The percentage of cells in the tissue or tumor core is printed. Scale bar = 100 μm. (B) Example image and quantification of cell invasion with an H2B-mCherry internal control. Confocal image (I) and 3D projection (II) of mixed H2B-mCherry (magenta) and H2B-mNeonGreen (green) expressing cells. (III) Histogram of invasion distances show overlapping invasion patterns for the H2B-mCherry and H2B-mNeonGreen expressing cells with a similar genetic background. Scale bar = 100 μm.

58. After exporting the data on invasion distance from every brain slice, copy the columns with the invasion distances into a single file for further statistical analysis. The number of invading and non-invading cells can be determined and a histogram can be made to show the distribution of cells within the brain slice (Figure 5).

Methods video S3. Imaris analysis steps to quantify the distribution of glioma nuclei in the tumour core and invasive front, related to steps 52–58

### Determining proliferation rate of glioma cells in the organotypic brain slices: BrdU assay

**Timing: 6 h**

Alternative to the whole-mount immunostaining method, after fixation the *ex vivo* organotypic brain slices can be further sectioned with a cryostat. This allows for immunostainings with antibodies that are not compatible with the whole-mount immunostaining method. In the next sections we describe how a BrdU assay can be performed on glioma cells injected into organotypic brain slices, to determine the level of proliferation of these cells.

59. Prepare the organotypic brain slices and inject the glioma cells as described before, up to step 30 of this protocol.
60. On the 7^th^ day after injection, place a drop of 50 μL 40 mM 5-Bromo-2′-deoxyridine (BrdU) in NSC-medium on top of the lateral ventricles containing the glioma cells and incubate at 37°C in a humidified 5% CO_2_ incubator for 6 h (Figure 6A).

**Figure 6:**
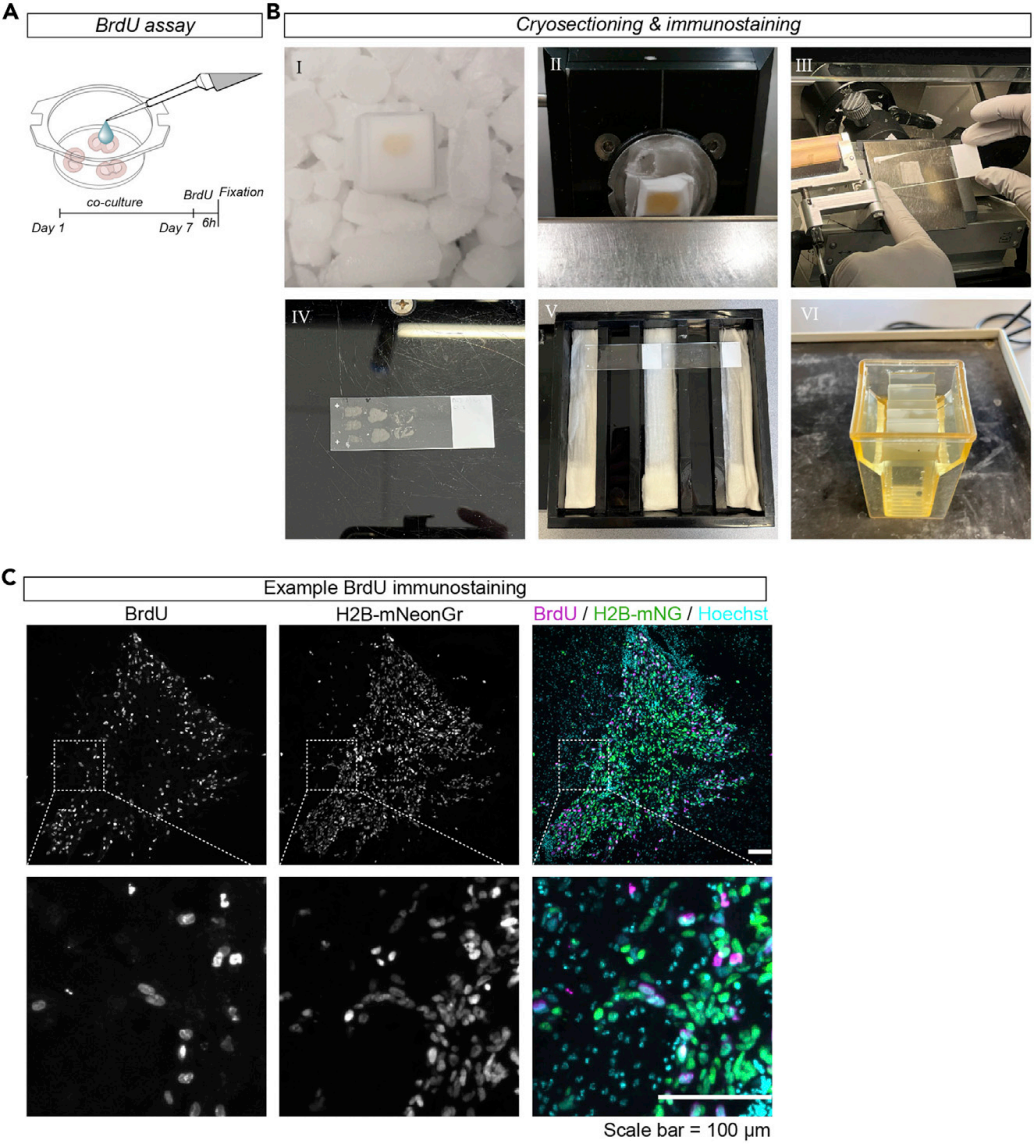
BrdU assay to determine proliferation (A) Schematic representation of BrdU assay. (B) Example images of cryo-sectioning & immunostaining steps described in step 65(I), step 66 (II), step 67a (III), step 67b (IV), step 74 (V) and step 75 (VI) of the step-by-step method details. Pictures were taken with a smartphone camera. (C) Sectioned organotypic brain slice injected with H2B-mNeonGreen (green) cells, stained with BrdU (magenta) and counterstained with Hoechst (cyan). Scale bar = 100 μm. Images were taken with an AxioScope A1 epifluorescent microscope.
61. After 6 h of incubation with BrdU, fixate and wash the tissue as described in steps 31 and 32 of this protocol.

### Cryosectioning of fixated organotypic brain slices

**Timing: 3 days**

This section describes the cryosectioning procedure of the fixated organotypic brain slices incubated with BrdU.

62. Cut the membrane of the insert around the individual brain slices and transfer to an embedding mold.
63. Submerge the brain slices in 30% sucrose in PBS solution (pH = 7.2) and incubate at 4°C for 48 h. Upon incubation in the sucrose solution, the brain slices should have sunk to the bottom of the embedding mold.
64. Remove the sucrose solution and wash the slices once with PBS.
65. Add Tissue Tek to the brain slices and snap freeze by placing the embedding molds in 2-Methylbutan (−55°C) on dry-ice for 2 min until the Tissue Tek is frozen (Figure 6B-I).
66. Transfer the snap-frozen brain slices to a cryostat with a chamber temperature of −20°C and an objective temperature of −18°C (Figure 6B-II).
67. Use the cryostat to section the brain slices into 20 μm sections.
  a. After making a section, hover over the tissue with a SuperFrost Plus Adhesion microscope slide so the tissue will spontaneously and permanently attach to it (Figure 6B-III).
  b. Collect the tissue on different microscopy slides in a series of four or five (Figure 6B-IV).

***Note:*** Take time to orient the brain slice at the right angle relative to the knife of the cryostat, so that it matches the cutting surface of the vibratome sections.

68. Upon collection of all tissue, let the sections dry at room temperature (19°C–21°C) until the Tissue Tek has evaporated. Store the microscopy slides at −20°C or directly proceed to the immunofluorescence staining.

### BrdU Immunofluorence staining of tissue sections

**Timing: 2 days**

The section below describes the antigen retrieval and immunofluorescence procedure to immunostain BrdU-positive cells and the analysis steps to calculate the percentage of BrdU-positive cells in the cryosectioned tissue slices.

69. Take out the glass microscope slides from −20°C and air dry in an incubation box (Figure 6B-V).
70. Wash the tissue three times with PBS to get rid of the remaining traces of Tissue Tek.
71. Perform antigen retrieval on the tissue sections.
  a. Cover the tissue sections with HCl 2 N and incubate at 37°C for 30 min.
  b. Incubate the tissue sections in Borate Buffer at room temperature (19°C–21°C) for 15 min.
  c. Wash three times in PBS (pH = 7.2) with 0.25% Triton-X100.
72. Dab the edges of the glass microscope slides carefully with a tissue paper without touching the brain tissue. Delimit the area around the brain tissue with a hydrophobic pen to minimize antibody volume.
73. Incubate the tissue sections in blocking buffer (materials and equipment) at room temperature (19°C–21°C) for 90 min.
74. Incubate the tissue sections with BrdU primary antibodies:
  a. Add the antibody to blocking buffer diluted 1:1 in PBS. Prepare 100–300 μL per microscopy slide, dependent on the size of the delimited area. In our experiments, we used rat anti-BrdU (1:500).
  b. Place the microscopy slides with tissue sections in a humidified chamber by placing wet paper tissues in the incubation box (Figure 6B-V).
  c. Add the primary antibody mix to the microscopy slides and incubate at 4°C overnight (min 12 h).
75. Wash the tissue sections three times for 5–10 min with PBS by transferring the microscopy slides to a slide staining jar on a shaker (Figure 6B-VI).
76. Incubate the tissue sections with secondary antibodies:
  a. Add the secondary antibodies and Hoechst (1: 1 000) or an alternative nuclear stain to PBS with 0.25% triton. Prepare 100–300 μL per microscopy slide, dependent on the size of the delimited area. In our experiments, we used donkey anti-rat-AF647 (1:1 000) secondary antibodies.
  b. Place the microscopy slides in a humidified chamber.
  c. Add the secondary antibody mix and incubate at room temperature (19°C–21°C) for 1 h, protected from light.
77. Wash the tissue sections three times with PBS, protected from light.
78. Mount the glass microscope slides with a coverslip.
  a. Remove the PBS by holding the glass microscope slide vertically on a paper tissue.
  b. Place three drops of FluorSave reagent or an alternative mounting solution on one side of the microscopy slide.
  c. Place a 25 × 60 mm coverslip with one edge on top of the mounting solution drops and slowly lower it to spread the mounting solution over the microscopy slide. Avoid making bubbles.
  d. Remove excess mounting solution by placing the microscope slide on a paper tissue.
79. Let the microscope slide dry overnight (min 12 h) at room temperature (19°C–21°C) before imaging with epifluorescence microscopy or alternative imaging methods.
80. Calculate the percentage of BrdU-positive nuclei by using the Analyze Particles function (Analyze > Analyze Particles) or using the manual cell counter plugin (Plugin > Analyze > Cell Counter > Cell Counter).

## Expected outcomes

By combining *ex vivo* organotypic invasion model with whole-mount immunostaining and tissue clearing, the invasion patterns of labeled glioma cells of interest can be reconstructed. In our hands, this methodology was used to visualize perivascular invasion of H2B-mNeonGreen labeled U251-MG cells along laminin-stained blood vessels. By using vimentin-staining, the cytoskeleton of individually invading glioma cells was visualized (Figure 3B). Additionally, mouse astrocytes can be stained using antibodies against glial fibrillary acidic protein (GFAP, Figure 3B-IV), to study interactions between astrocytes and glioma cells. Although the GFAP antibody can recognize GFAP in human U251-MG cells and in mouse astrocytes, differences in expression levels and the presence of a fluorescent reporter in the human cells allows to distinguish the two cell types.

Successful visualization of invasion patterns is also expected when cytosolic labels are used (Figure 3B-IV), however, the analysis steps described in this protocol are optimized for nuclear labeling. Using these analysis steps, the number of invading cells and the distribution of cells in the tissue can be quantified (Figure 5A). To cope with variation between samples, an internal control can be included in the experimental design, in which control cells with a different fluorescent label are co-injected with the experimental cells (step 22, Figures 4C and 4D). We used this strategy to compare the invasive capacities of U251-MG cells with different cytoskeletal GFAP networks (Uceda-Castro et al., 2022).

After BrdU-incorporation, cryosectioning of the *ex vivo* organotypic brain slices, and BrdU-staining, nuclear staining of BrdU-positive cells are anticipated (Figure 6C). By dividing the number of BrdU-positive nuclei by the total number of nuclei, the percentage of mitotic cells within a specific time-frame can be calculated.

## Limitations

The co-culture method described in this protocol allows investigation of glioma cell invasion and proliferation in a brain-like environment where brain geometrical cues and ECM compositions are maintained. We did not investigate the host-cell – glioma cell interaction in this model, as the effect of the culture conditions may be a limitation for these types of questions. For instance, cell death of mouse brain cells is expected, and this will alter the physiology of the surrounding brain cells.

A limitation of the whole-mount immunostaining protocol is that not all primary antibodies fully penetrate the tissue or have a good signal-to-noise ratio. In our hands, the BrdU antibody was not compatible with the whole-mount immunostaining protocol, explaining why we describe staining with this antibody on cryo-sectioned material. For clearing of the whole-mount immunostained tissue, we selected the RapiClear clearing method. There are multiple tissue clearing methods developed and published, each with their advantages, disadvantage, and suitability for types of tissues (Silvestri et al., 2016). The absence of shrinkage and the non-laborious protocol makes RapiClear a suitable clearing method for organotypic brain slices, however, a disadvantage is that we observed a difference in signal intensity over the Z-axis, indicating suboptimal clearing. Therefore, this method is less suitable for quantitative measurements of signal intensity between samples. Also, during the analysis of distribution of nuclei using the Imaris software, there might be a selection bias during the quality threshold filtering (step 54e) of nuclei that are more superficial. Since we observe that most cells migrate in the XY direction, we do not expect that this bias will have major impacts on the outcome of the analysis, nevertheless, using an internal control (step 22) can be considered to cope with this bias. Lastly, the tissue-clearing steps described in this protocol are incompatible with cellular dyes, therefore visualization of cells and tissues is dependent on fluorescent proteins and immunostainings.

## Troubleshooting

### Problem 1

Steps 16 and 17: The vibratome slicing leads to irregular brain slices, curled up tissue, or loosening of the mounted brain.

### Potential solution

Solution 1: Optimize the amount of glue used when mounting the mouse brain to the specimen disk (step 11). Too little glue can cause partial detachment of the brain during the slicing, resulting in irregular and curled up slices. When too much glue is used, it can cover the part of the brain that will be collected during the slicing and disturb the vibratome slicing. Aim for a drop of glue the size of a pea.

Solution 2: Replace the razor blade. The razor blade can be re-used multiple times, but after a couple of re-uses, it will become blunt and disturb the slicing.

Solution 3: When the tissue has become a little loose, it can help to place a brush directly behind the tissue to avoid movement.

### Problem 2

Step 26: Injection of the cells leads to a flow of cells on top of the tissue, or underneath the tissue.

### Potential solution

Injection of the cells works best when the cells can fill up pre-existing free space like the ventricles or the space created with the syringe itself. In the XY axis, place the syringe in the tissue in the same line as the longest axis of the ventricle to stimulate a smooth flow of cells. In the XZ axis, place the syringe right below the surface of the tissue so that there is sufficient space available for the cells and direct injection on the membrane of the cell culture insert is avoided. Another solution is to decrease the speed of injection. Lastly, in our experience, there is more overflow of cells when the slices are washed right before injection, as a layer of liquid remains on top of the slice that promotes the flow of cells. We, therefore, do not recommend washing the top of the brain slices right before injections.

### Problem 3

Step 28: The injected cells look deformed or unhealthy after injection.

### Potential solution

When the syringe is improperly washed with PBS after cleaning with ethanol, the cells may look deformed after injection and will not invade the tissue. To avoid this, wash the syringe repeatedly with PBS before taking up cells and do not clean the syringe with ethanol in-between the injections.

### Problem 4

Step 44: Weak signal-to-noise ratio of the fluorescent protein expressed by the cells.

### Potential solution

The brain slice tissue can give rise to a high background, which can lead to a weak signal-to-noise ratio when the intensity of the fluorescent protein is low. Consider using a different fluorescent protein with better intensity or include a fluorescent protein-specific antibody during the whole-mount immunostaining protocol.

### Problem 5

Step 67: Only half of the tissue is collected during the cryosectioning.

### Potential solution

The angle of the tissue relative to the knife is essential to collect a section of the entire brain slice during cryosectioning. Take more time in fine-tuning the angle of the knife after making the first slices to be sure that the tissue is sectioned correctly.

## Resource availability

### Lead contact

Further information and requests for resources and reagents should be directed to and will be fulfilled by the lead contact, Elly M. Hol, e.m.hol-2@umcutrecht.nl.

### Materials availability

This study did not generate unique reagents.

## Data Availability

This study did not generate datasets.

## References

[bib1] Bekkouche B.M.B., Fritz H.K.M., Rigosi E., O’Carroll D.C. (2020). Comparison of transparency and shrinkage during clearing of insect brains using media with tunable refractive index. Front. Neuroanat..

[bib2] Belle M., Godefroy D., Dominici C., Heitz-Marchaland C., Zelina P., Hellal F., Bradke F., Chédotal A. (2014). A simple method for 3D analysis of immunolabeled axonal tracts in a transparent nervous system. Cell Rep..

[bib3] Drost J., van Jaarsveld R.H., Ponsioen B., Zimberlin C., van Boxtel R., Buijs A., Sachs N., Overmeer R.M., Offerhaus G.J., Begthel H. (2015). Sequential cancer mutations in cultured human intestinal stem cells. Nature.

[bib4] Humpel C. (2015). Organotypic brain slice cultures: a review. Neuroscience.

[bib5] Markovic D.S., Glass R., Synowitz M., Rooijen N.v., Kettenmann H. (2005). Microglia stimulate the invasiveness of glioma cells by increasing the activity of metalloprotease-2. J. Neuropathol. Exp. Neurol..

[bib6] Pencheva N., de Gooijer M.C., Vis D.J., Wessels L.F.A., Würdinger T., van Tellingen O., Bernards R. (2017). Identification of a druggable pathway controlling glioblastoma invasiveness. Cell Rep..

[bib7] Preibisch S., Saalfeld S., Tomancak P. (2009). Globally optimal stitching of tiled 3D microscopic image acquisitions. Bioinformatics.

[bib8] Uceda-Castro R., van Asperen J.V., Vennin C., Sluijs J.A., van Bodegraven E.J., Margarido A.S., Robe P.A.J., van Rheenen J., Hol E.M. (2022). GFAP splice variants fine-tune glioma cell invasion and tumour dynamics by modulating migration persistence. Sci. Rep..

[bib9] Schneider C.A., Rasband W.S., Eliceiri K.W. (2012). NIH Image to ImageJ: 25 years of image analysis. Nat. Methods.

[bib10] Sigma Aldrich (2021). https://www.sigmaaldrich.com/MX/en/technical-documents/technical-article/genomics/cloning-and-expression/blue-white-screening.

[bib11] Silvestri L., Costantini I., Sacconi L., Pavone F.S. (2016). Clearing of fixed tissue: a review from a microscopist’s perspective. J. Biomed. Opt..

[bib12] Sliwa M., Markovic D., Gabrusiewicz K., Synowitz M., Glass R., Zawadzka M., Wesolowska A., Kettenmann H., Kaminska B. (2007). The invasion promoting effect of microglia on glioblastoma cells is inhibited by cyclosporin A. Brain.

[bib13] www.sunjinlab.com (2017). Instruction – SUNJIN LAB. https://www.sunjinlab.com/instruction/.

